# Early Life Modifiable Exposures and Their Association With Owner Reported Inflammatory Bowel Disease Symptoms in Adult Dogs

**DOI:** 10.3389/fvets.2021.552350

**Published:** 2021-02-01

**Authors:** Manal Hemida, Kristiina A. Vuori, Robin Moore, Johanna Anturaniemi, Anna Hielm-Björkman

**Affiliations:** ^1^Department of Equine and Small Animal Medicine, Faculty of Veterinary Medicine, University of Helsinki, Helsinki, Finland; ^2^Department of Nutrition and Clinical Nutrition, Faculty of Veterinary Medicine, Beni-Suef University, Beni-Suef, Egypt

**Keywords:** chronic enteropathies, canine, diet, microbiome, gut, immune, prenatal

## Abstract

**Background:** Inflammatory bowel disease (IBD) is an idiopathic multifactorial disease in humans and dogs, usually assigned to the interactions between genes, gut microbiota, diet, environment, and the immune system. We aimed to investigate the modifiable early life exposures associated with IBD in dogs.

**Materials and Methods:** The study data was extracted from the validated owner-reported DogRisk food frequency questionnaire. This was a cross-sectional questionnaire-based study that tested 21 different early life dietary and environmental, demographic and genetic variables for their association with IBD or not, in adult dogs. A total of 7,015 dogs participated in this study. The study covered early life periods; prenatal, neonatal, early, and late postnatal periods. Two feeding patterns, a non-processed meat-based diet (NPMD) and an ultra-processed carbohydrate-based diet (UPCD) were studied. Data was analyzed using logistic regression analysis with a backward stepwise deletion.

**Results:** From the final models we found that the NPMD during early and late postnatal periods were significantly associated with lower IBD risk later in life. The UPCD during the same periods was associated with a higher risk of IBD incidence. Also, the maternal diet during the neonatal period showed a non-significant trend of lower IBD risk in the offspring with the NPMD and a higher IBD risk with the UPCD. Additionally, the normal body weight of puppies during the first 6 months of age was associated with a lower risk of IBD in adulthood while, slim puppies associated significantly with IBD in adulthood. From the non-modifiable background variables, we identified the maternal history of IBD as the strongest risk factor for later incidence of IBD. Furthermore, male dogs were twice as likely to develop IBD as female dogs were.

**Conclusions:** It is reassuring for owners to know that they themselves can have an impact on their dog's health. A high-fat, low-carbohydrate NPMD exposure during early life, and a normal body condition in puppyhood were significantly associated with less IBD in adult dogs. The opposite was true for UPCD exposure and abnormal body condition score in 6 month old puppies.

## Introduction

Canine inflammatory bowel disease (IBD) is a group of chronic idiopathic enteropathies in dogs characterized by persistent and/or recurrent gastrointestinal symptoms (1–3). In this study we will refer to these enteropathies as IBD. The disease usually affects middle-aged dogs with no gender predisposition (4, 5); although intermittent symptoms have been noted even in puppies < 6 months of age (5). Clinical phenotypes of IBD have been identified and used to define specific forms of IBD with varying breed predisposition [(1, 5, 6), Table 1]. To date, the etiopathogenesis of idiopathic IBD, both in humans and canines, is not fully understood. Current literature supports the concept that IBD is usually assigned to the interactions between genetics, gut microenvironment (dietary microbiota and diet composition), and the host immune system (1, 2). The genetic component of the disease has been recognized using both genome-wide association and candidate gene approaches in humans (14, 15) and dogs (6, 16, 17).

**Table 1:** Inflammatory bowel disease prone breeds.

**IBD phenotypes**	**IBD prone breeds**
Inflammatory bowel disease	Akita^1^, Bernese mountain dog^1^, Dalmatian^1^, English setter^1^, German shepherd^1^^,^^2^^,^^7^, Golden retriever^1^^,^^2^, Irish setter^1^, Pointer^1^, Rottweiler^1^^,^^2^, Soft-coated wheaten terrier^1^^,^^3^^,^^7^, Labrador retriever^2^, Border colli^2^, Boxer^2^^,^^7^, Staffordshire bull terrier^2^, Cocker spaniel^2^, West highland white terrier^2^, Weimaraner^2^, Jack Russell terrier^2^, Basenjis^3^^,^^7^, Mixed breeds^2^, French bull dog^3^, Doberman pinscher^3^, Mastiff^3^, Alaskan malamute^3^, Shar pei^7^
Intestinal malabsorption	Akita^1^, Basenji^1^, Chinese Shar pei^1^, Chow chow^1^, French bull dog^1^, Irish setter^1^, Old English sheep dog^1^, Peruvian inca orchid^1^, Rottweiler^1^, Shiloh shepherd^1^, Soft-coated wheaten terrier^1^
Gluten-sensitive enteropathy	Irish setter^1^^,^^2^
Ulcerative colitis	Akbash^1^, Boxers^1^^,^^4^^,^^5^^,^^7^, German shepherd^1^, Skye terrier^1^, French bull dog^5^^,^^7^, Mastiff^5^^,^^7^, Alaskan malmalute^5^^,^^7^, Dobermann pinscher^5^^,^^7^
Tylosin responsive diarrhea	Irish setter^6^, Basenji^6^, Lundehund^6^, Yorkshire terrier^6^, German shepherd^6^, Boxer^6^, French bull dog^6^, Shar-pei^6^, Rottweiler^6^, Soft-coated wheaten terrier^6^
Antibiotic responsive diarrhea	German shepherds^8^

1–8 *cited from Dodds (7), Kathrani et al. (8), Cerquetella et al. (2), Davies et al. (9), Stokes et al. (10), Westermarck et al. (11), Hall (12), Hall (13) respectively*.

Mounting evidence from human epidemiological studies suggests that it is wise to focus on exploring the role that early life exposures have on influencing the gut microbiome and immune modulation, as it in turn can modify the disease risk (18–21). Several theories encourage identifying the role of environmental stimulants, including diet, in triggering the inflammatory response. The most prominent of them is the hygiene hypothesis which states that an increased frequency of immune disorders can be attributed to a reduction in enteric microbiota during early life. This has been presumed to be due to exaggerated sanitation, which results in an untrained, and therefore malfunctioning, immune system (22, 23). The newborn immune maturation is mainly driven by the early life exposure to microbes (24). The gut microbiota is the central source of the postnatal microbial exposure (25). The early life diet has a profound effect on the neonate gut microbiota and thereby also on immune regulation (26). Dietary patterns are a fundamental part of a healthy lifestyle and diets can influence gut microbial ecosystems, promoting gut health in dogs and humans. Recently, diet composition has been shown to substantially impact the abundance and modulation of gut microbiome in dogs and humans (27–34). Moreover, the diet processing; whether the diet is offered as a non-processed/raw diet or as an ultra-processed diet, has been observed to impact human and canine health (35, 36).

Research regarding the role of the early diet on IBD incidence in small animals is scarce. One study on cats analyzed the role that early life events and diet had on gastrointestinal symptoms that developed later in life (37). They found that when owners reported diarrhea, vomiting, and/or decreased consumption of commercial diets before their cat was 16 weeks of age, it was also associated with gastrointestinal symptoms occurring at least twice between 6 and 30 months of age (37). Although the disease is frequently presented at animal clinics, there is no data on the true prevalence of IBD in dogs (4). Currently there are no studies that identify early life exposures that might act as risk factors for chronic IBD development in dogs. Furthermore, the possible influence of a non-processed meat based diet and an ultra-processed carbohydrate based diet on the prevalence of IBD in dogs has not been previously assessed. Only one canine study found a significant risk of developing gastrointestinal diseases if a dog had previously suffered from a parvovirus infection (38). Therefore, it is important to investigate modifiable early life risk factors for canine IBD that might have an impact on future immune system stimulation. The main aim of the present study was to investigate possible associations of modifiable early life exposures, dietary and environmental, with owner-reported IBD incidence in later life. In addition, we aimed to test previous known risk factors of the disease, such as maternal history, gender, and breed.

## Materials and Methods

### The Questionnaire and Study Population

The study data was extracted from the DogRisk food frequency questionnaire (FFQ) data. This FFQ is an online validated ongoing questionnaire (39) http://www.ruokintakysely.fi/. It has been available online for dog owners since 2009, when it was launched by us at the University of Helsinki, Finland. The whole questionnaire is in Finnish language. As mentioned elsewhere (36, 39, 40) the FFQ includes 50 questions generating 1,332 data points, reported by the dog owners. It aims to gather information on the owner's dog's diets and lifestyle at different time points throughout its life, as well as data on the dog's health conditions, background, and demographic information. In addition to gathering information on the dog itself, it also contains several questions concerning the dogs' mothers' early maternal diets and their diseases. The FFQ has an ethical approval (29.4.2016) from the University of Helsinki Viikki campus ethical board.

**Figure 1:**
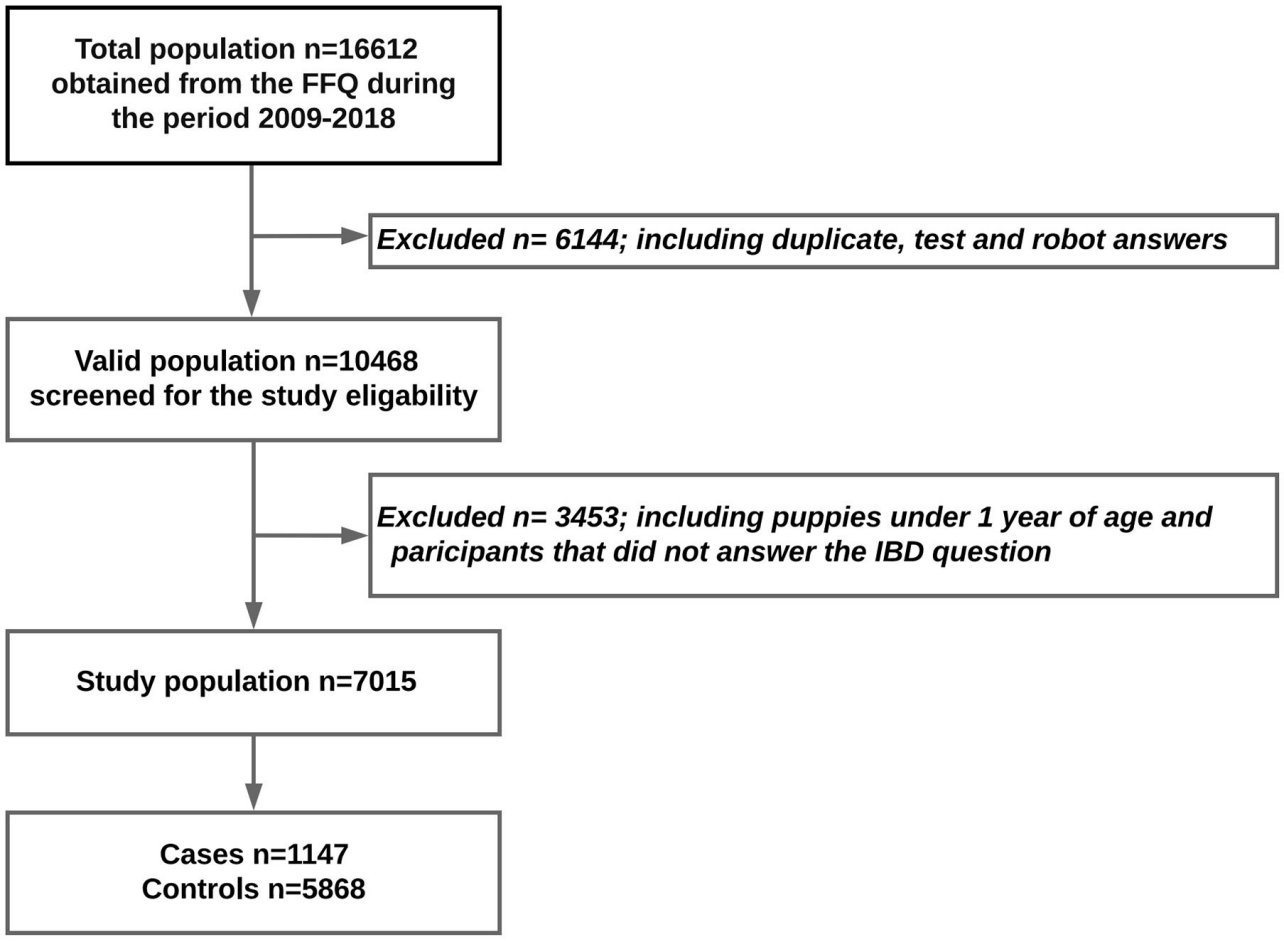
Flow chart of the study population.

The questionnaire received 16,559 responses between 2009 and 2019. The dogs under 1 year of age were excluded from this study in order to avoid reverse causality. Also, participants who had not answered the question about whether their dogs had been suffering from IBD or not were excluded. Otherwise, all breeds and both sexes were included. After all questionnaire test answers, robot answers, and duplicates had been removed, 7,015 participants were eligible for the study (Figure 1).

### Study Design and Tested Variables

This is a once-answered, and in that respect cross-sectional, questionnaire-based study asking about multiple exposures at different time points, therefore also making it longitudinal. This study has been carried out to investigate the association between early life modifiable exposures (dietary and environmental) and the incidence of owner-reported IBD later in life. In addition, we tested the associations between non-modifiable genetic and demographic variables and IBD incidence. The study tested only one dependent categorical dichotomous variable, concerning IBD, for association with 21 different independent categorical and continuous variables (Table 2). The dependent variable obtained by responding either “Yes” or “No” to the question: “Has your dog suffered from inflammatory bowel disease (IBD), chronic bowel symptoms, chronic bowel ‘allergies’?” The study cases were obtained when the owners responded “Yes,” while those who responded “No” were chosen as study controls.

**Table 2:** Frequencies of the tested variables in the cases, controls, and total study cohort.

**Variables**	**Categories**	**Inflammatory bowel disease %(n)**		
		**Cases, %**	**Controls, %**	**Total, %**
		**(*n =* 1,147)**	**(*n =* 7,015)**	**(*n =* 7,015)**
***Non- modifiable; genetic and demographic factors***				
Maternal History of IBD	Mothers without IBD	90.0 (260)	98.8 (2,135)	97.7 (2,395)
	Mothers with IBD	10.0 (29)	1.2 (27)	2.3 (56)
Dog breed	IBD prone breeds	55.5 (562)	50.4 (2,583)	51.2 (3,145)
	IBD non-prone breeds	44.5 (450)	49.6 (2,546)	48.8 (2,996)
Dog gender	Males	60.3 (675)	43.4 (2,480)	46.2 (3,155)
	Females	39.7 (444)	56.6 (3,232)	53.8 (3,676)
Dog color	White >50%	21.8 (240)	20.0 (1,107)	20.3 (1,347)
	White <50%	78.2 (862)	80.0 (4,441)	79.7 (5,303)
Dog age, years (mean ± SD)^*^		5.10 ± 3.06	5.04 ± 3.12	5.05 ± 3.11
***Modifiable factors***				
**Prenatal period (pregnancy)**				
Maternal gestation diet	NPMB	6.0 (22)	9.1 (178)	8.6 (200)
	UPCD	94.0 (346)	90.9 (1,788)	91.4 (2,134)
Was the mother dewormed during/just before pregnancy?	Yes	95.9 (446)	96.2 (2,717)	96.2 (3,163)
	No	4.1 (19)	3.8 (107)	3.8 (126)
Was the mother vaccinated during/just before pregnancy?	Yes	59.5 (150)	48.7 (830)	50.1 (980)
	No	40.5 (102)	51.3 (873)	49.9 (975)
**Neonatal period (lactation)**				
Maternal lactation diet	NPMB	6.2 (21)	9.2 (172)	8.7 (193)
	UPCD	93.8 (319)	90.8 (1,706)	91.3 (2,025)
**Early postnatal period (puppy 1–2 months of age)**				
Puppy's first solid diet	NPMD	5.9 (22)	10.3 (198)	9.6 (220)
	UPCD	94.1 (353)	89.7 (1,720)	90.4 (2,073)
Frequency of outdoor activity	Many times/day	51.4 (331)	60.0 (2,105)	58.6 (2,436)
	Once/day	15.5 (100)	15.7 (552)	15.7 (652)
	A few times/week	14.4 (93)	11.5 (405)	12.0 (498)
	A few times/month	7.3 (47)	5.0 (176)	5.4 (223)
	Not at all	11.3 (73)	7.8 (273)	8.3 (346)
Rest, hours/day (mean ± SD)^*^		15.99 ± 3.97	15.98 ± 3.80	15.98 ± 3.82
Type of flooring	Slippery flooring	9.7 (66)	10.9 (399)	10.7 (465)
	Non-slippery flooring	26.3 (179)	27.4 (1,006)	27.2 (1,185)
	Dirt flooring	5.9 (40)	7.5 (275)	7.2 (315)
	Newspaper flooring	30.0 (204)	27.8 (1,023)	28.2 (1,227)
	Carpet flooring	28.1 (191)	26.5 (973)	26.7 (1,164)
Body condition score	Obese puppy	14.6 (109)	14.6 (573)	14.6 (682)
	Normal weight puppy	75.1 (561)	75.9 (2,977)	75.8 (3,538)
	Slim puppy	10.3 (77)	9.5 (371)	9.6 (448)
**Late postnatal period (puppy 2–6 months)**				
Puppy diet	NPMD	15.1 (45)	22.2 (310)	20.9 (355)
	UPCD	84.9 (254)	77.8 (1,088)	79.1 (1,342)
Outdoor activity, hours/day	< 0.5	1.2 (9)	2.2 (89)	2.1 (98)
	0.5–1.0	28.3 (219)	25.5 (1,019)	26.0 (1,238)
	1.0–2.0	51.8 (401)	51.5 (2,057)	51.6 (2,458)
	> 2.0	18.7 (145)	20.7 (826)	20.4 (971)
Rest, hours/day (mean ± SD)^*^		14.52 ± 3.21	14.43 ± 3.28	14.45 ± 3.27
Type of flooring	Slippery flooring	23.4 (191)	24.5 (1,040)	24.3 (1,231)
	Non-slippery flooring	24.9 (203)	25.1 (1,064)	25.0 (1,267)
	Dirt flooring	1.0 (8)	2.0 (85)	1.8 (93)
	Newspaper flooring	0.5 (4)	0.3 (11)	0.3 (15)
	Carpets flooring	12.0 (98)	13.5 (572)	13.2 (670)
Body condition score	Obese puppy	7.3 (59)	6.4 (266)	6.5 (325)
	Normal weight puppy	61.6 (495)	69.0 (2,883)	67.8 (3,378)
	Slim puppy	31.1 (250)	24.7 (1,032)	25.7 (1,282)
Was the puppy vaccinated 2–4 times under 1 year of age?	Yes	98.1 (1,050)	98.7 (5,493)	98.6 (6,543)
	No	1.9 (20)	1.3 (72)	1.4 (92)
Was the puppy dewormed 2–10 times under 1 year of age?	Yes	98.9 (1,018)	98.8 (5,303)	98.8 (6,321)
	No	1.1 (11)	1.2 (64)	1.2 (75)

*(n): the number of dogs, IBD, inflammatory bowel disease; NPMD, non-processed meat based diet; UPCD, ultra-processed carbohydrate based diet*.

* *Scale variables presented as (mean ± SD)*.

**Figure 2:**
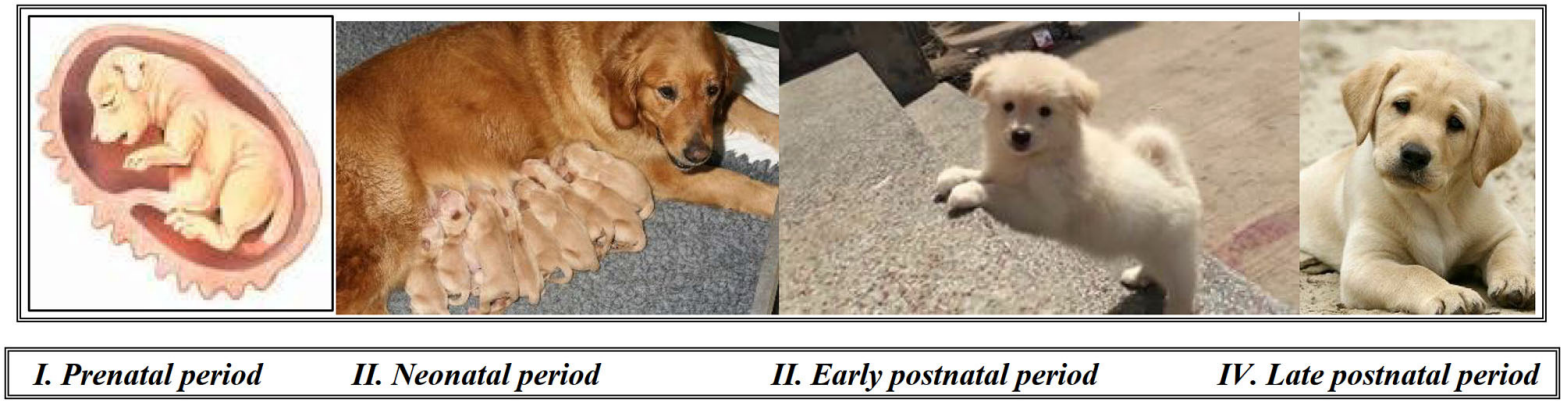
Pathway of the study variables at different time points. Prenatal period image adapted from https://dogs.lovetoknow.com/wiki/Canine_Gestation, neonatal period image adapted from https://www.yorkbeach.co.uk/puppies/daisy_2016.html, early postnatal period image adapted from https://i.ytimg.com/vi/8nt7M12CTa0/maxresdefault.jpg, and late postnatal period image adapted from https://www.pets4homes.co.uk/pet-advice/what-to-expect-of-your-puppy-at-4-to-6-months-old.html.

We analyzed four early life periods, prenatal (pregnancy period), neonatal (the 1st 3–4 weeks of life, i.e., the lactation period), early postnatal (from 1 to 2 months of age) and late postnatal periods (from 2 to 6 months of age) as shown in Figure 2.

The modifiable early life variables that were tested covered nutritional, environmental and lifestyle factors. We compared two common feeding patterns, the non-processed meat-based diets (NPMD) and the ultra-processed carbohydrate-based diets (UPCD) during the following early life periods: the maternal diet during pregnancy, the maternal diet during lactation, the young puppy first solid diet and the puppy diet in the period between 2 and 6 months of age. The NPMD is a diet rich in fresh animal proteins and fats (red meat, poultry, fish, organs, bones, lard, fish oils etc.) and low in carbohydrates but including raw vegetables, fruits or berries for micronutrients and vegetal fibers. The NPMD is either commercial or prepared at home by chopping, grating, mixing and freezing the ingredients. The UPCD refers to commercially produced extruded kibble. Typically, 40–60% of the contents of the UPCD are processed carbohydrates (mostly grains, rice, potatoes etc. but also some vegetable pulp). The two diets; NPMD and UPCD, exhibit different macronutrient profiles but may also differ in many other ways.

We tested four environmental and lifestyle factors during the early and late postnatal periods including outdoor activity, rest hours, type of flooring, and the body condition score. The body condition score (BCS) was a 5-category scale where the owner could choose between 1 = very slim, 2 = slim, 3 = normal, 4 = round/fat, and 5 = obese.

The questions about vaccination and deworming programs of the pregnant dams and puppies under 1 year of age were also tested for the association with the dependent variable. The question regarding the dog's mother was: “Did you vaccinate/deworm the dam while she was pregnant or just before?” The question regarding the puppy asked whether or not it had received 2–4 vaccinations before the age of 1 year? The answers of these questions were, “yes,” “no” or “I do not know/I do not remember.” Only the yes and no answers were used in the analyses.

The final five questions regarded the non-modifiable genetic and demographic variables, namely: maternal history of IBD, dog age, dog gender, dog color (specifically wanting to know the amount of white in the coat) and if the dog breed is prone to IBD or not prone to IBD (Table 1). The variable of IBD prone breed or not, was gathered from the literature (Table 1).

### Statistical Methods

The categorical and discrete variables were presented as frequencies *n* (%) using cross-tabulation for the cases, controls and the total study population, while the continuous variables were summarized as means and standard deviations (mean ± SD). The disease prevalence was calculated using cross-tabulation as ratios of the diseased dogs in the total population and within each age group. Moreover, the prevalence was calculated after stratifying the FFQ cohort for gender, maternal history, and disease predisposition in the different breeds.

The association of the tested independent variables with IBD symptoms in adult dogs was calculated using logistic regression analyses. Firstly, the variables were analyzed using univariate logistic regression and the variables with a *p* < 0.2 were used for final modeling. Five final models were created using the backward stepwise regression method. The first model included the demographic non-modifiable variables, the other four models contained the early life modifiable exposures during prenatal life, neonatal life, early postnatal life and late postnatal life, respectively. The five models were adjusted for age and the statistical significance was considered for *p-*values lower than 0.05 (^*^), 0.01 (^**^), and 0.001 (^***^). To help the veterinarian or nutritionist reader make an informed choice regarding the type of food that they usually prescribes in their practice, we report our regression analysis using two dummy variables that were created for the dichotomous variables instead of one. The missing values were not imputed. To test the fitness of the regression models an Omnibus test *p-*value should be lower than 0.05, a Hosmer and Lemeshow test *p-*value should be larger than 0.05 and the Nagelkerke's R should be as big as possible (41, 42). The statistical analyses were performed using SPSS version 25. The visualization of odds ratios was carried out using the forest plot package (43) in R software version 3.5.1 (44).

## Results

### Inflammatory Bowel Disease Prevalence (IBD) in the Finnish DogRisk Food Frequency Questionnaire (FFQ) Population

**Figure 3:**
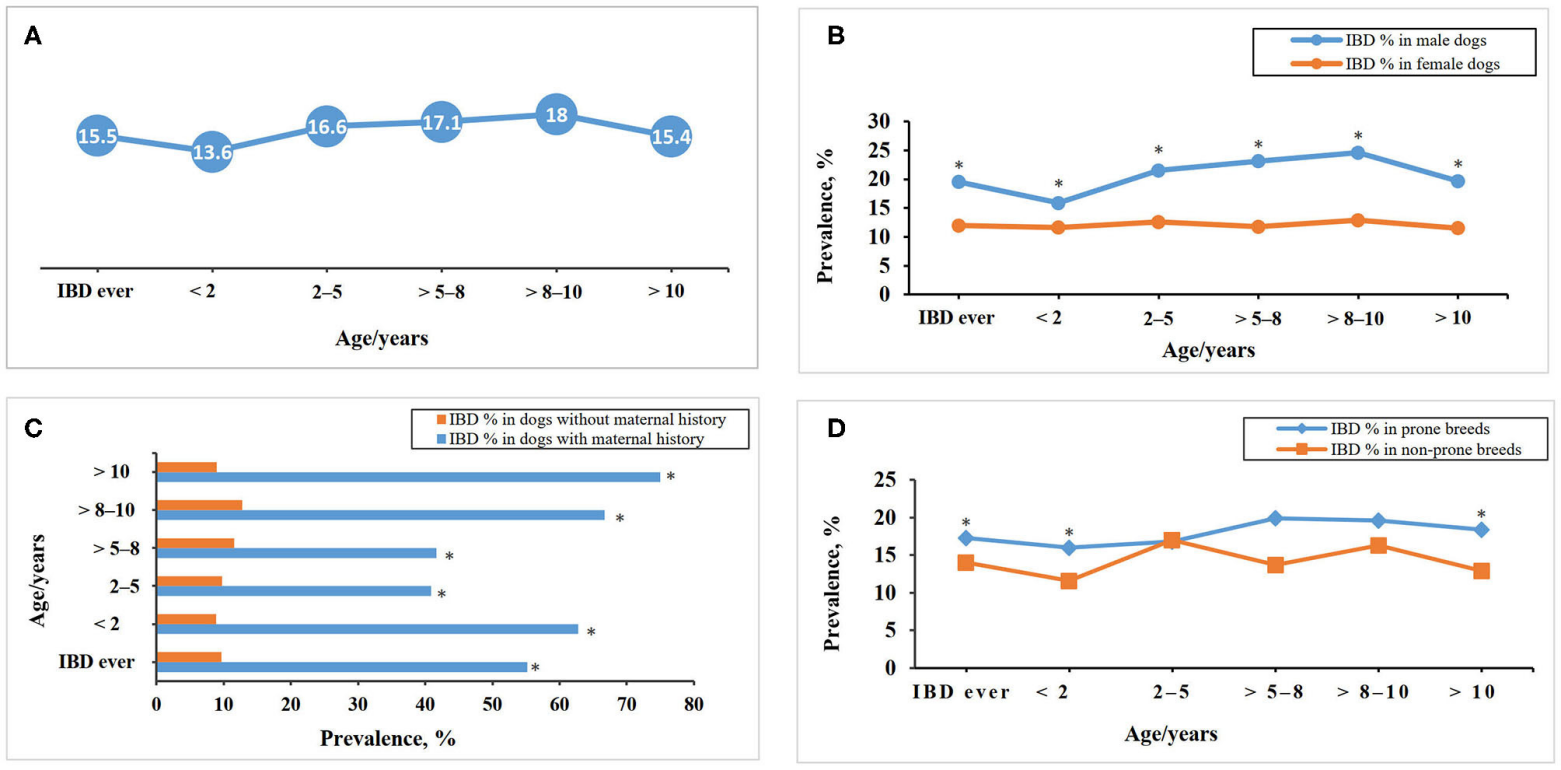
Inflammatory bowel disease prevalence within different age groups in the DogRisk food frequency questionnaire population, *n* = 10468, **(A)** and when the population was stratified for gender **(B)**, maternal history **(C)**, and disease predisposition in the different breeds **(D)**. *The difference between the two groups within each age group is significant at *P* < 0.05.

The prevalence of IBD in the FFQ population differs between the age groups and when the population was stratified for gender, history of maternal disease, white color coat ratio, and IBD prone breeds as shown in Figure 3.

### Variables Characteristics

The distribution of the tested variables within the cases, controls and the total study cohort are presented in Table 2 as frequencies (%) and numbers (n) for the categorical variables, while the scale variables are presented as means ± SDs (Table 2).

### Regression Analysis

The univariate logistic regression analysis showed 10 significant associations (*p* < 0.05). Additionally, six variables (*p* < 0.2) were also included in the final models (Table 3).

**Table 3:** Associations of early life covariates with inflammatory bowel disease in dogs based on univariate logistic regression analyses.

**Covariates**	**Crude effect estimates**		**Multivariate models**
	**cOR (95% CI)**	***P-*value**	
***Non- modifiable; genetic and demographic factors***			
***Maternal history of IBD***			Included in model I
Non-IBD mothers vs. IBD mothers	0.113 (0.066–0.195)	<0.001^***^	
IBD vs. non-IBD mothers	8.820 (5.141–15.131)	<0.001^***^	
***Dog breed***			Included in model I
Non-IBD prone vs. IBD prone	0.812 (0.709–0.930)	0.003^**^	
IBD prone vs. non-IBD prone	1.231 (1.075–1.410)	0.003^**^	
***Dog gender***			Included in model I
Female vs. male	**0.505 (0.443–0.575)**	<0.001^***^	
Male vs. female	1.981 (1.739–2.258)	<0.001^***^	
***Dog color***			Included in model I
> 50% white coat vs. < 50%	1.117 (0.954–1.307)	**0.169**	
< 50% white coat vs. > 50%	0.895 (0.765–1.048)	**0.169**	
***Dog age^§^***	1.007 (0.987–1.027)	0.505	Included in model I
***Modifiable factors***			
***I. Prenatal period maternal factors***			
***Mother's diet during pregnancy***			Included in model II
NPMD vs. UPCD	0.639 (0.404–1.009)	**0.055**	
UPCD vs. NPMD	1.566 (0.991–2.474)	**0.055**	
***Was the mother dewormed during/just before pregnancy?***			Not included
Yes vs. no	0.924 (0.562–1.521)	0.757	
No vs. yes	1.082 (0.657–1.780)	0.757	
***Was mother vaccinated during/just before pregnancy?***			Included in model II
Yes vs. no	1.547 (1.182–2.024)	0.001^**^	
No vs. yes	0.647 (0.494–0.846)	0.001^**^	
***II. Neonatal period (0–1 month of age)***			
***Mother's diet during lactation***			Included in model III
NPMD vs. UPCD	0.653 (0.409–1.043)	**0.075**	
UPCD vs. NPMD	1.532 (0.959–2.447)	**0.075**	
***III. Early postnatal period (1–2 months of age)***			
***Puppy's first solid diet***			Included in model IV
NPMD vs. UPCD	0.541 (0.343–0.853)	0.008^**^	
UPCD vs. NPMD	1.847 (1.172–2.912)	0.008^**^	
***Frequency of outdoor activity***			Included in model IV
Many times/day vs. not at all	0.588 (0.443–0.780)	<0.001^***^	
Once/day vs. not at all	0.677 (0.485–0.947)	0.023^*^	
A few times/week vs. not at all	0.859 (0.610–1.210)	0.384	
A few times/month vs. not at all	0.999 (0.661–1.509)	0.995	
***Type of flooring***			Included in model IV
Slippery vs. dirt flooring	1.137 (0.746–1.734)	0.550	
Non-slippery vs. dirt flooring	1.223 (0.847–1.767)	0.283	
Newspaper vs. dirt flooring	1.371 (0.953–1.973)	**0.089**	
Carpets vs. dirt flooring	1.350 (0.936–1.946)	**0.109**	
***Rest, hours/day***	1.001 (0.958–1.045)	0.977	Not included
***Body condition Score***			Not included
Slim vs. normal weight puppies	1.101 (0.848–1.431)	0.469	
Obese vs. normal weight puppies	1.009 (0.807–1.263)	0.934	
***IV. Late postnatal period (2–6 months of age)***			
***Puppy diet***			Included in model V
NPMD vs. UPCD	0.622 (0.442–0.875)	0.006^**^	
UPCD vs. NPMD	1.608 (1.143–2.262)	0.006^**^	
***Outdoor activity, hours/day***			Included in model V
0.5–1 vs. < 0.5	2.125 (1.054–4.284)	0.035^*^	
1–2 vs. < 0.5	1.928 (0.963–3.858)	**0.064**	
> 2 vs. < 0.5	1.736 (0.855–3.524)	**0.127**	
***Rest, hours/day***	1.008 (0.983–1.034)	0.528	Not included
***Type of flooring***			Included in model V
Slippery vs. dirt flooring	1.951 (0.930–4.094)	**0.077**	
Non-slippery vs. dirt flooring	2.027 (0.967–4.250)	**0.061**	
Newspaper vs. dirt flooring	3.864 (0.997–14.973)	**0.051**	
Carpets vs. dirt flooring	1.820 (0.855–3.876)	**0.120**	
More than two types vs. dirt flooring	2.249 (1.078–4.690)	0.031^*^	
***Body condition score***			Included in model V
Slim vs. normal weight puppies	1.411 (1.193–1.669)	<0.001^***^	
Obese vs. normal weight puppies	1.292 (0.959–1.740)	**0.092**	
***Was the puppy vaccinated 2–4 times under 1 year of age?***			Included in model V
Yes vs. no	0.688 (0.417–1.134)	**0.143**	
No vs. yes	1.453 (0.882–2.395)	**0.143**	
***Was the puppy dewormed 2–10 times under 1 year of age?***			Not included
Yes vs. no	1.117 (0.587–2.125)	0.736	
No vs. yes	0.895 (0.471–1.703)	0.736	

*cOR, Crude odds ratio; CI, Confidence intervals; IBD, Inflammatory bowel disease; NPMD, Non-processed meat based diet; UPCD, Ultra-processed carbohydrate based diet; vs., versus*.

* *p < 0.05*,

** *p < 0.01*,

*** *p < 0.001*,

*Bolded values indicate p < 0.2*,

§ *scale variable measured in years*.

**Figure 4:**
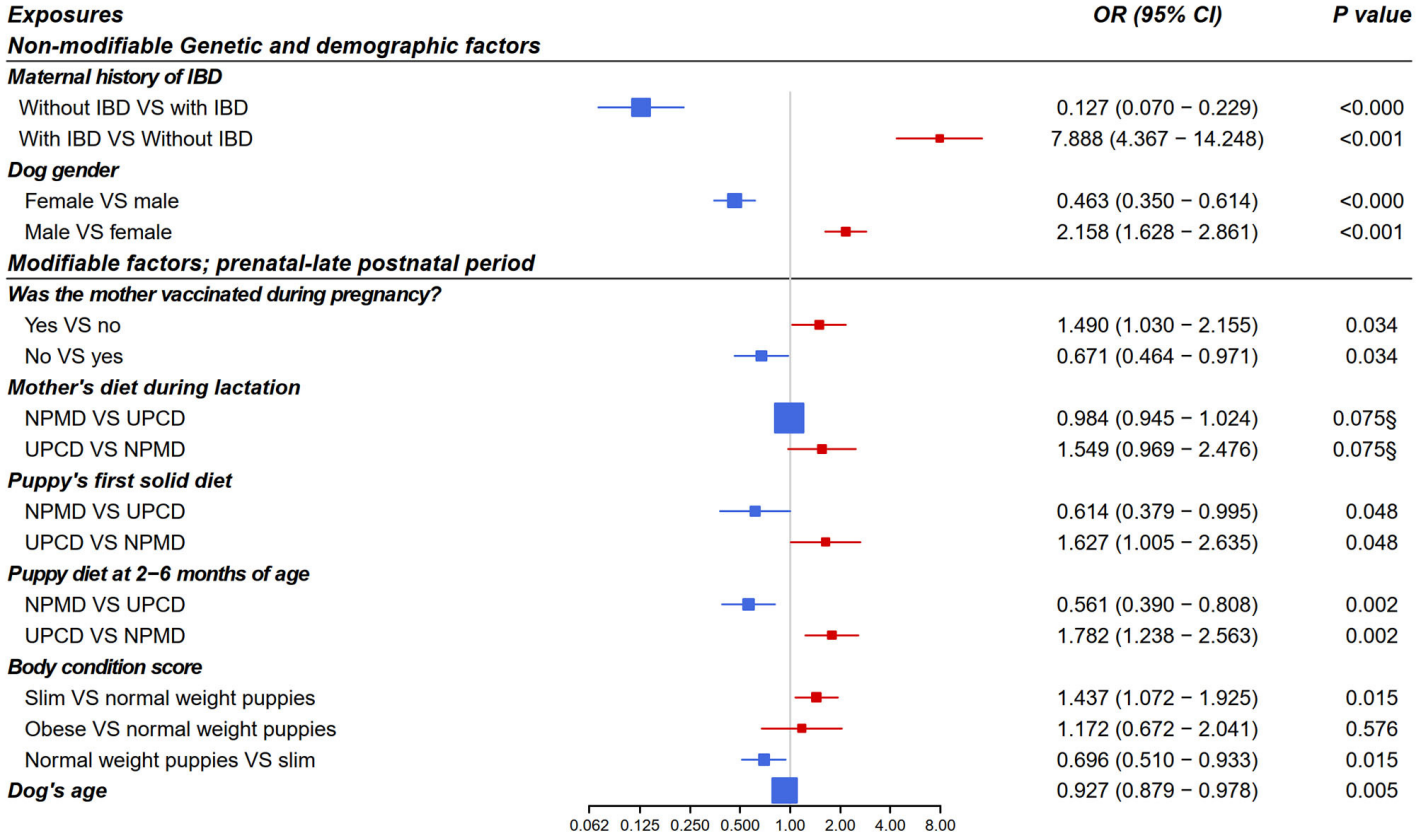
Forest plot of odds ratios for the association of early life exposures and inflammatory bowel disease in adult dogs based on multivariate logistic regression analysis (*n* = 7015, adjusted for dog age). Included dogs for each model: model 1 (*n* = 1976), model 2 (*n* = 1022), model 3 (*n* = 2218), model 4 (*n* = 1830), and model 5 (*n* = 1532). Modifiable exposures included models from 2 to 5 as following model 2: prenatal period, model 3: neonatal period, model 4: early postnatal period, model 5: late postnatal period. OR, odds ratio; CI, confidence interval; IBD, inflammatory bowel disease; NPMD, non-processed meat based diet; UPCD, ultra-processed carbohydrate based diet; VS, versus. ^§^non-significant *p* > 0.05.

From the multivariate logistic regression models, four early-life exposures showed a significant association with canine IBD incidence in adulthood and one early-life exposure showed a non-significant “trend” as shown in Figure 4. The final models' odds ratios for the associations of the non-modifiable and the early life modifiable factors with IBD in adult dogs are presented in Figure 4. A *p* < 0.05 and an OR > 1 means increased risk for IBD while *p* < 0.05 and OR < 1 means decreased risk.

## Discussion

A growing trove of literature regarding genes and the microbiome confirmed by data from human epidemiological studies advocates the importance of early life exposures in modulating the risk of IBD incidence (18, 45–48). However, the impact that early life environmental and nutritional exposures have on IBD risk in dogs has not been previously examined. This is the first study to investigate the association between modifiable early life exposures and the incidence of IBD in adult dogs. Exploring the most pertinent early life exposures provides clues to the etiopathogenesis of this complex disease and allows for the development of a primary prevention strategy for IBD in dogs. At the same time, as a dog's pregnancy and early life is shorter than a human's, but otherwise similar in both terms of environment and diet, this study might also provide insight regarding the prevention of human IBD. Our results are divided into the non-modifiable factors that cannot be altered, and the modifiable factors, where the owners' choices can have an impact on their dog's future health.

### Non-modifiable Exposures and How They Related to Owner Reported IBD

Our analysis of the non-modifiable background related factors showed that the maternal history of IBD was directly associated with IBD incidence in the offspring in later life. To the best of our knowledge, the family history of IBD in dogs and the risk of the disease morbidity in the offspring later, has not been demonstrated before. The role that the family disease history has on the offspring's subsequent IBD development in adulthood, has been studied in several human studies (18, 49–53). Our findings are similar to a human study, where they found that the greatest risk factor for IBD was having one or more affected first-degree relative (52). In the case of having two parents with IBD, the long-term risk of developing the disease in the offspring was over 30% (53). The increased risk of IBD in dogs with a maternal history of it supports the proposed genetic component of the disease (6). However, other theories exist: Freud et al. (54) concluded that long-term morbidity of pediatric diseases in the offspring up to 18 years of age (such as cardiovascular, endocrinal, respiratory, hematological, neurological, urinary, and gastrointestinal diseases) were not affected by maternal IBD during pregnancy. This suggests that genetics alone cannot explain the increased prevalence of IBD, instead it synergizes with other potential risk factors, especially diet (1, 2).

In the present study, the male dogs exhibited a higher risk of developing IBD than females did. Similarly, a Japanese study that studied the prevalence of chronic enteropathy in dogs through using an insurance-based population found that there was a slightly higher disease prevalence in males compared to female dogs (55). On the other hand, some studies have reported that there is no gender predisposition of IBD development in dogs (5, 56). As IBD is often used as an umbrella term for different diseases, the different types of IBD and the different breeds studied may be the reason for this discrepancy between different studies.

In our study, there was a significant association between the incidence of IBD and the dog's age. We found that the highest IBD prevalence within different age groups was in middle-aged dogs, from 5 to 10 years of age. This finding is in accordance with one study (5), and with anecdotal knowledge that IBD is mainly a disease of middle-aged dogs.

The incidence of IBD showed a significant difference between the IBD-prone and non-prone breed cohorts (Figure 3). Breed predisposition has been recognized for IBD [(7, 8), Table 1]. Gluten sensitive enteropathy has been reported with Irish setters (7, 8), while ulcerative colitis has been found to be most common in boxers (7, 9, 10, 12). Chronic enteropathy or protein-losing enteropathy is the most common form of IBD. This form can affect many breeds and mixed-breed dogs (2, 7, 8, 12). German shepherd dogs are more prone to develop antibiotic responsive diarrhea (13), especially tylosin responsive diarrhea (11).

### Modifiable Exposures and How They Related to Owner Reported IBD

Next we present the modifiable prenatal exposures that were significantly associated with IBD prevalence in adulthood. Surprisingly, the maternal vaccination during or just before pregnancy was significantly associated with more IBD in the offspring in adulthood, while not vaccinating the dam was associated with less IBD in the offspring at adult age. Literature on the risks of prenatal exposure to vaccines and incidence of IBD later in dogs remains limited. However, our finding is in line with studies of adjuvants such as aluminum salts and mercury-containing compounds such as thimerosal, that have been reported to be involved in the development of inflammatory disorders (57, 58) and stimulation of the immune system in humans (59). A human study testing the effect of childhood vaccination and risk of IBD later found an association between an early life measles vaccination and the risk of IBD later (60), while two other studies found no association between measles vaccines in early life and the risk of IBD (61, 62). Moreover, a meta-analysis showed that the majority of childhood vaccinations against different infectious diseases do not increase the risk for subsequent IBD development (63). We also analyzed the associations between early life puppy vaccinations and IBD and found that these vaccinations had no association with IBD. As there results are controversial and our data only suggestive, we recommend further studies looking at this before any conclusions are drawn.

From the modifiable postnatal exposures, the maternal diet during the lactation/neonatal period appeared to be an important modifiable factor in our study, although it did not reach significance in our final models. Evidence proposes that the maternal gut microbiome may be translocated intracellularly to the mammary glands through the systemic circulation (64). This has been studied in humans, where the authors concluded that the maternal diet in the neonatal period alters the gut microbiome of the offspring, which subsequently modulates the risk of related diseases through breastfeeding (65). The role of the maternal diet during lactation has been confirmed to shape the lifelong health of the newborn human child (66). However, the direct impact of the maternal diet on the milk microbiome during pregnancy and lactation was not established in our study. Future research is needed to test how different feeding patterns in dogs during pregnancy and lactation affects the neonate gut microbiota diversity.

During the early and late postnatal periods, there were significant negative associations between the NPMD and incidence of IBD for the same dogs later in life. These findings are in accordance with several studies, which stated that a raw meat-based diet stimulated the growth of a balanced gut microbiome in healthy dogs which improved their gut function in comparison to dogs fed an extruded dry food (28, 30, 36, 67). Our observations are in accordance with the hygiene hypothesis, which states that the more microbial exposures in the early life, the more developed immune system in adulthood (22, 23). This confirms the role of the NPMD in developing the immune system when given in the early life (14, 68).

**Figure 5:**
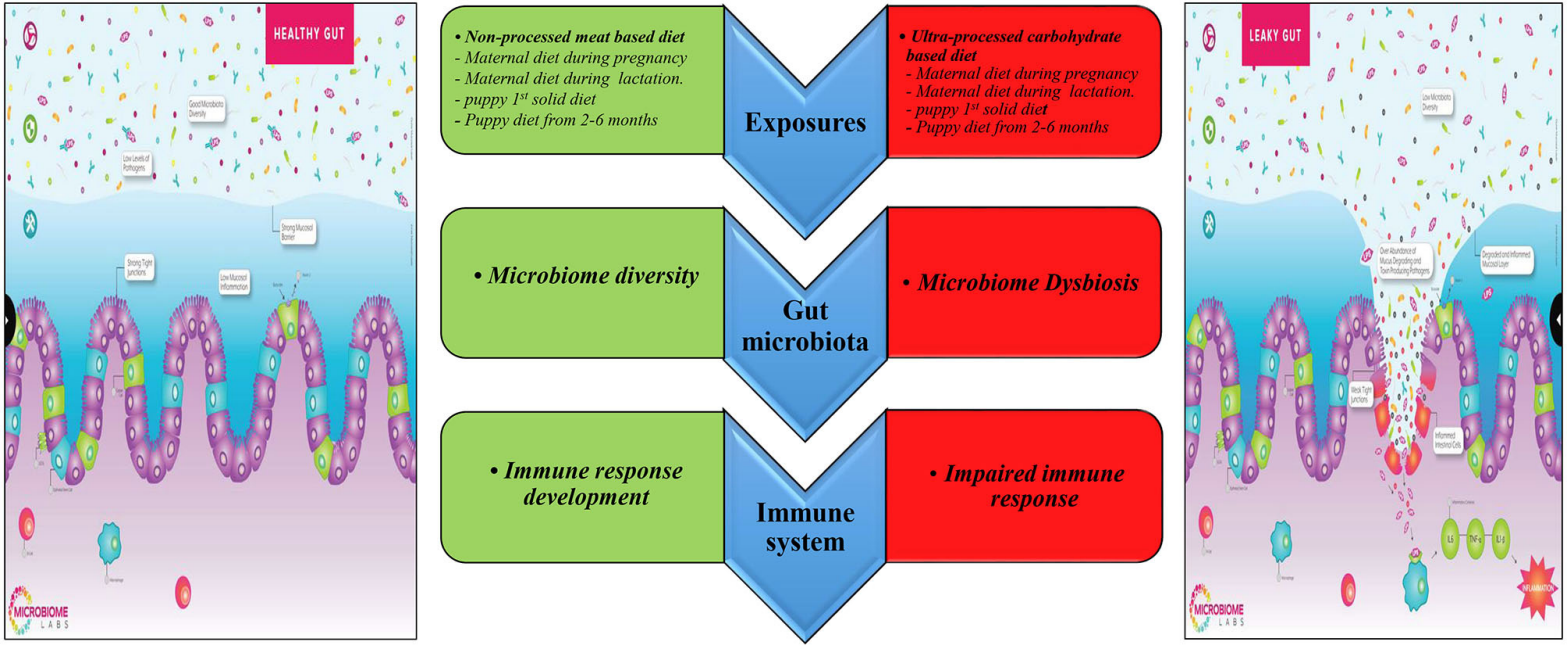
Prospective mechanisms underlining the relationship between early diet and IBD in adult dogs. Edited from the Microbiome Labs (https://microbiomelabs.com/).

Besides the interaction between diet, gut microbiome, and the immune system discussed above (Figure 5), there are additional factors that may explain the effect of the early life diet on the offspring's risk of IBD in adulthood. First, the maternal and postnatal diet can permanently modify the epigenetic programming in the newborn during its formative early life. A recent review suggested that the individual becomes resistant or susceptible to diseases by altering inflammatory molecular pathways and immunity via epigenetic modification (69). However, the underlying mechanisms are not clear. Evidence suggests that the maternal gut microbiome can affect the neonate gut microbiome by causing a particular epigenetic signature that can influence the intestinal barrier's properties against inflammatory diseases (69).

Next we discuss the effects that distinct dietary components may have on gut health and physiology. The UPCD contains a high ratio of (carbohydrate and gluten-rich) processed cereal, and hence may increase the dogs' risk of developing gluten sensitive enteropathy (70–72). Furthermore, in mouse studies, the authors found that the consumption of diets rich in refined carbohydrates increased intestinal dysbiosis, permeability, and inflammation (73, 74). As sugars are highly absorbable and carbohydrates are chains of short sugar molecules, a high-carbohydrate diet may cause a high sugar concentration in the intestinal lumen. This in turn may supply excessive energy for the microbiota and hence lead to intestinal bacterial overgrowth (75). The UPCD also contains a low dietary fiber content. Dietary fibers, also called prebiotics, are non-digestible carbohydrates responsible for maintaining normal intestinal homeostasis through encouraging bacterial diversity, protecting mucosal barriers, and increasing the synthesis of short chain fatty acids (76, 77). Furthermore, the processing methods, which the UPCD or their ingredients, are exposed to, such as rendering, milling, fermentation, heat treatment, and extrusion negatively affects the bioavailability of key minerals and vitamins present in the diet (78, 79). Feed additives, e.g., dietary emulsifiers which are used to improve kibble texture have been found to contribute to the increased incidence of inflammatory diseases both by reducing the gut microbiota diversity and by reducing the thickness of the mucus layer (80). Since dogs have no requirement for carbohydrates (81), this underlines the fact that carbohydrates should not be a main ingredient in a dog's diet (82). As carnivores, dogs have evolved to eat fresh meat-based diets rich in animal proteins, fats, and animal fibers (83, 84). A canine study showed that a raw meat-based diet was both highly palatable and highly digestible when compared to an extruded diet. Moreover, although including lots of fats and proteins, the NPMD decreased blood triglyceride levels, maintained normal serum chemistry and high fecal quality, and altered the fecal microbiota and metabolite concentration (85).

The body condition score during the late puppyhood period revealed that there was a significantly negative association between IBD development and dogs with normal body weight, while puppies with a lower body weight associated positively with developing the disease.

Prior research on IBD shows that there is an association between increase in obesity in the population and IBD prevalence (86). Obesity is a known perpetual factor of systemic low-grade inflammation (87), generating a pro-inflammatory state and immune dysregulation in obese puppies (88). Furthermore, a review also established an association between increased IBD morbidity and malnutrition (89). Additionally, a study found that young IBD patients have been demonstrated to have weight loss prior to diagnosis (90), where 57% of patients with Crohn's disease and 51% of patients with ulcerative colitis exhibited a significantly low body mass index prior to diagnosis. The association of a low body condition score during puppyhood with IBD incidence later in life maybe a reflection of active undiagnosed disease at young age. The loss in weight prior to or at the time of the disease diagnosis is attributed to many factors. As IBD is an inflammatory disease, it stimulates catabolism, alternates metabolic hormones levels, increases the gastrocolic reflux leading to loss of appetite, and causes nutrient malabsorption (90). Although incidences of IBD in puppies < 6 months old have been recorded (5), it remains unclear whether the loss in body weight during puppyhood is a cause or a consequence of IBD. Further research is needed to understand the physiological disturbance underlying the association between the body condition score and active IBD.

### Limitations and Strengths of the Study

The present study has some limitations. Firstly, the study design is an owner-reported cross-sectional study which makes it prone to recall bias. In order to overcome this limitation, a thorough questionnaire of the activity and dietary habits of 2,000 different aged Finnish dogs was conducted prior to this FFQ (not published). Based on these results, we constructed this DogRisk internet-based FFQ where all of the foods that were mentioned by owners in the first questionnaire were included as multiple choice questions in the new one. Two empty rows were also given to the owner, if there still would have been things missing. It is considered better to give options to owners that they can choose from when they see them written, that that they should remember all things by themselves. Another limitation is that the FFQ is based on frequencies and not quantity. Also, due to the lack of details regarding the ingredients of the food variables, we were not able to examine the nutrient profiles of the diets. Moreover, the study cases and controls were reported by the owners. We did not ask the owners for a veterinary visit wrap-up, including the diagnosis. To some extent, this has been overcome by validating different types of diseases from the FFQ in a previous validation study (39). Another limitation is that the data has a lot of missing values. This was overcome by retaining a reasonable sample size (*n* = 7,015).

The present study also has strengths. It is the first study to investigate early life environmental and nutritional exposures and their association with IBD incidence in dogs. It is also the first study to investigate the associations between genetic and demographic variables on IBD incidence in dogs. A wide range of covariates were covered and validated data was used (39), both which favor the study's validity, reliability and reproducibility. Furthermore, our data included information about four early life periods, pre- and postnatal, which covered 8 months of the dog's life starting from conception till the age of 6 months. Finally, reverse causality was considered and addressed in this study when choosing the age limits of the cases and controls.

## Conclusions and Recommendations

In conclusion, our study identified many modifiable early life risk and protective factors for canine IBD incidence in adulthood. We conclude that a raw feeding pattern, which typically features a moderate protein, high fat, and low carbohydrate macronutrient profile, during neonatal, early and late postnatal periods, as maternal and puppy diets, was associated with a decreased risk of IBD later in life. Conversely, a dry extruded feeding pattern, which mostly includes a moderate protein, low fat, and high carbohydrate macronutrient profile, during the same periods, was associated with an increased risk of developing IBD later in life. In addition, maternal vaccination during or just prior to pregnancy was significantly associated with a higher risk of IBD incidence in the offspring later. Furthermore, a normal body condition score was associated with a decreased risk of IBD development, while being abnormal in weight (very lean) was associated with an increased risk of IBD at adult age. As foretold, the identified non-modifiable risk factors from the current study such as dogs with a history of maternal IBD, male dogs, middle-aged dogs, and dogs from breeds prone to IBD development, were all associated with an increased risk of IBD prevalence. Our novel findings regarding the modifiable risk factors provide clues for further research in the disease prevention. The study findings suggest a causal relationship but does not prove it. Future prospective longitudinal dietary intervention studies are needed to confirm our findings, as well as to develop primary strategies for IBD prevention in dogs.

## Data Availability Statement

Being funded by commercial sources has not altered our adherence to Frontiers policies on sharing data and materials. The data is still used for theses and will be totally disclosed later. However, for research purposes the data can be obtained upon request from the authors: anna.hielm-bjorkman@helsinki.fi.

## Ethics Statement

The animal study was reviewed and approved by Ethical approval (29.4.2016) from the University of Helsinki Viikki campus ethical board. Written informed consent was obtained from the owners for the participation of their animals in this study.

## Author Contributions

MH and AH-B planned, designed, and drafted the study. AH-B organized the database. MH and AH-B performed the data extraction and did the statistical analysis together with KV who created the figures from statistical software. All authors wrote sections of the manuscript and edited it, contributed to manuscript revision, read, and approved the submitted version.

## Conflict of Interest

The authors declare that the research was conducted in the absence of any commercial or financial relationships that could be construed as a potential conflict of interest.

## Abbreviations

IBD: inflammatory bowel disease
NPMD: non-processed meat based diet
UPCD: ultra-processed carbohydrate based diet
FFQ: food frequency questionnaire.

## References

[1] Simpson KW Jergens AE. Pitfalls and progress in the diagnosis and management of canine inflammatory bowel disease. Vet Clin North Am Small Anim Pract. (2011) 41:381–98. 10.1016/j.cvsm.2011.02.003 21486642

[2] Cerquetella M Spaterna A Laus F Tesei B Rossi G Antonelli E. Inflammatory bowel disease in the dog: differences and similarities with humans. World J Gastroenterol. (2010) 16:1050–6. 10.3748/wjg.v16.i9.1050 20205273 PMC2835779

[3] Jergens AE. Inflammatory bowel disease: current perspectives. Vet Clin North Am Small Anim Pract. (1999) 29:501–21. 10.1016/S0195-5616(99)50032-6 10202800

[4] Dandrieux JRS Mansfield CS. Chronic enteropathy in canines: prevalence, impact and management strategies. Vet Med. (2019) 10:203–14. 10.2147/VMRR.S162774 31828025 PMC6902862

[5] Yogeshpriya S Veeraselvam M Krishnakumar S Arulkumar T Jayalakshmi K Saravanan M Technical review on inflammatory bowel disease in dogs and cats. Int J Sci Environ Technol. (2017) 6:1833–42.

[6] Peiravan A Bertolini F Rothschild MF Simpson KW Jergens AE Allenspach K. Genome-wide association studies of inflammatory bowel disease in German shepherd dogs. PLoS ONE. (2018) 13:e0200685. 10.1371/journal.pone.0200685 30028859 PMC6054420

[7] Dodds WJ Guide to Congenital and Deritable Disorders in Dogs. hsVma. Davis, CA: The Humane Society Veterinary Medical Association (2011). Available online at: https://www.hsvma.org/assets/pdfs/guide-to-congenital-and-heritable-disorders.pdf

[8] Kathrani A Werling D Allenspach K. Canine breeds at high risk of developing inflammatory bowel disease in the south-eastern UK. Vet Rec. (2011) 169:635. 10.1136/vr.d5380 21896567

[9] Davies DR O'Hara AJ Irwin PJ Guilford WG. Successful management of histiocytic ulcerative colitis with enrofloxacin in two Boxer dogs. Aust Vet J. (2004) 82:58–61. 10.1111/j.1751-0813.2004.tb14643.x 15088960

[10] Stokes JE Kruger JM Mullaney T Holan K Schall W. Histiocytic ulcerative colitis in three non-boxer dogs. J Am Anim Hosp Assoc. (2001) 37:461–5. 10.5326/15473317-37-5-461 11563445

[11] Westermarck E Skrzypczak T Harmoinen J Steiner JM Ruaux CG Williams DA. Tylosin-responsive chronic diarrhea in dogs. J Vet Intern Med. (2005) 19:177–86. 10.1111/j.1939-1676.2005.tb02679.x 15822561

[12] Hall EJ Breed-specific intestinal disease. In: World Small Animal Veterinary Association 29th World Congress Proceedings. Rhodes (2004).

[13] Hall EJ. Antibiotic-responsive diarrhea in small animals. Vet Clin Small Anim. (2011) 41:273–86. 10.1016/j.cvsm.2010.12.004 21486636

[14] Andersena V Olsenc A Carbonneld F Tjønnelandc A Vogel U. Diet and risk of inflammatory bowel disease. Dig Liver Dis. (2012) 44:185–94. 10.1016/j.dld.2011.10.001 22055893

[15] Jostins L Ripke S Weersma RK Duerr RH McGovern DP Hui KY. Host-microbe interactions have shaped the genetic architecture of inflammatory bowel disease. Nature. (2012) 491:119–24. 10.1038/nature11582 23128233 PMC3491803

[16] Kathrani A Lee H White C Catchpole B Murphy A German A. Association between nucleotide oligomerisation domain two (Nod2) gene polymorphisms and canine inflammatory bowel disease. Vet Immunol Immunopathol. (2014) 161:32–41. 10.1016/j.vetimm.2014.06.003 25017709

[17] Karlsson EK Baranowska I Wade CM Salmon HNH Zody MC Anderson N. Efficient mapping of mendelian traits in dogs through genome-wide association. Nat Genet. (2007) 1321–8. 10.1038/ng.2007.10 17906626

[18] van der Sloot KWJ Weersma RK Dijkstra G. Development and validation of a web-based questionnaire to identify environmental risk factors for inflammatory bowel disease: the Groningen IBD Environmental Questionnaire (GIEQ). J Gastroenterol. (2019) 54:238–48. 10.1007/s00535-018-1501-z 30109418 PMC6394725

[19] Ananthakrishnan AN. Epidemiology and risk factors for IBD. Nat Rev Gastroenterol Hepatol. (2015) 12:205–17. 10.1038/nrgastro.2015.34 25732745

[20] van der Sloot KJW Amini M Peters V Dijkstra G Alizadeh BZ. Inflammatory bowel diseases: review of known environmental protective and risk factors involved. Inflamm Bowel Dis. (2017) 23:1499–509. 10.1097/MIB.0000000000001217 28777099

[21] Koloski NA Jones M Weltman M Kalantar J Bone C Gowryshankar A. Identification of early environmental risk factors for irritable bowel syndrome and dyspepsia. Neurogastroenterol Motil. (2015) 27:1317–25. 10.1111/nmo.12626 26202154

[22] Strachan DP. Hay fever, hygiene, and household size. BMJ. (1989) 299:1259–60. 10.1136/bmj.299.6710.1259 2513902 PMC1838109

[23] Klement E Lysy J Hoshen M Avitan M Goldin E Israeli E. Childhood hygiene is associated with the risk for inflammatory bowel disease: a population-based study. Am J Gastroenterol. (2008) 103:1775–82. 10.1111/j.1572-0241.2008.01905.x 18557710

[24] Nash MJ Frank DN Friedman JE. Early microbes modify immune system development and metabolic homeostasis-the “Restaurant” hypothesis revisited. Front Endocrinol. (2017) 8:349. 10.3389/fendo.2017.00349 29326657 PMC5733336

[25] Rodríguez JM Murphy K Stanton C Stanton C Ross RP Kober OI. The composition of the gut microbiota throughout life, with an emphasis on early life. Microb Ecol Health Dis. (2015) 26:26050. 10.3402/mehd.v26.26050 25651996 PMC4315782

[26] Parigi SM Eldh M Larssen P Gabrielsson S Villablanca EJ. Breast milk and solid food shaping intestinal immunity. Front Immunol. (2015) 6:415. 10.3389/fimmu.2015.00415 26347740 PMC4541369

[27] Deng P Swanson KS. Gut microbiota of humans, dogs and cats: current knowledge and future opportunities and challenges. Br J Nutr. (2015) 113:S6–17. 10.1017/S0007114514002943 25414978

[28] Sandri M Dal Monego S Conte G Sgorlon S Stefanon B. Raw meat based diet influences faecal microbiome and end products of fermentation in healthy dogs. BMC Vet Res. (2016) 13:65. 10.1186/s12917-017-0981-z 28245817 PMC5331737

[29] David LA Maurice CF Carmody RN Gootenberg DB Button JE Wolfe BE. Diet rapidly and reproducibly alters the human gut microbiome. Nature. (2013) 505:559–63. 10.1038/nature12820 24336217 PMC3957428

[30] Sandri M Sgorlon S Conte G Serra A Dal Monego S Stefanon B Substitution of a commercial diet with raw meat complemented with vegetable foods containing chickpeas or peas affects faecal microbiome in healthy dogs. Ital J Anim Sci. (2019) 18:1205–14. 10.1080/1828051X.2019.1645624

[31] Kerr KR Forster G Dowd SE Ryan EP Swanson KS. Effects of dietary cooked navy bean on the fecal microbiome of healthy companion dogs. PLoS ONE. (2013) 8:e74998. 10.1371/journal.pone.0074998 24040374 PMC3770567

[32] Middelbos IS Vester Boler BM Qu A White BA Swanson KS Fahey GCJ. Phylogenetic characterization of fecal microbial communities of dogs fed diets with or without supplemental dietary fiber using 454 pyrosequencing. PLoS ONE. (2010) 5:e9768. 10.1371/journal.pone.0009768 20339542 PMC2842427

[33] Panasevich MR Kerr KR Dilger RN Fahey GC Jr Guérin-Deremaux L Lynch GL. Modulation of the faecal microbiome of healthy adult dogs by inclusion of potato fibre in the diet. Brit J Nutr. (2015) 113:125–33. 10.1017/S0007114514003274 25418803

[34] Stercova E Kumprechtova D Auclair E Novakova J. Effects of live yeast dietary supplementation on nutrient digestibility and fecal microflora in beagle dogs. J Anim Sci. (2016) 94:2909–18. 10.2527/jas.2016-0584 27482677

[35] Monteiro CA Cannon G Moubarac JC Levy RB Louzada MLC Jaime PC. The UN decade of nutrition, the NOVA food classification and the trouble with ultra-processing. Public Health Nutr. (2018) 21:5–17. 10.1017/S1368980017000234 28322183 PMC10261019

[36] Hemida M Vuori KA Salin S Moore R Anturaniemi J Hielm-Björkman A. Identification of modifiable pre- and postnatal dietary and environmental exposures associated with owner-reported canine atopic dermatitis in Finland using a web-based questionnaire. PLoS ONE. (2020) 15:e0225675. 10.1371/journal.pone.0225675 32469869 PMC7259748

[37] Kathrani A Blackwell EJ Williams JL Gruffydd-Jones T Murray JK Hezzell M. Exploring early life events including diet in cats presenting for gastrointestinal signs in later life. Vet Rec. (2019) 185:144. 10.1136/vr.105040 31167836

[38] Kilian E Suchodolski JS Hartmann K Mueller RS Wess G Unterer S. Long-term effects of canine parvovirus infection in dogs. PLoS ONE. (2018) 13:e0192198. 10.1371/journal.pone.0192198 29547647 PMC5856261

[39] Roine J Uusitalo L Hielm-Bjorkman A. Validating and reliability testing the descriptive data and three different disease diagnoses of the internet-based DOGRISK questionnaire. BMC Vet Res. (2016) 12:30. 10.1186/s12917-016-0658-z 26897627 PMC4761135

[40] Anturaniemi J Uusitalo L Hielm-Björkman A. Environmental and phenotype-related risk factors for owner-reported allergic/atopic skin symptoms and for canine atopic dermatitis verified by veterinarian in a Finnish dog population. PLoS ONE. (2017) 12:e0178771. 10.1371/journal.pone.0178771 28570617 PMC5453595

[41] Dohoo I Martin W Stryhn H Hilbe J Anthony J Methods in Epidemiologic Research. Charlottetown: VER Inc (2012). p. 499–500.

[42] Hosmer DW Lemeshow S Applied Logistic Regression. 2nd ed New York, NY: Wiley (2000). 10.1002/0471722146

[43] Gordon M Lumley T Advanced forest plot using 'grid' graphics. R Package Version 1.9. (2019). Available online at: https://CRAN.R-project.org/package=forestplot

[44] R Core Team R: A Language and Environment for Statistical Computing. R Foundation for Statistical Computing. Vienna (2017). Available online at: https://www.R-project.org/

[45] Vedamurthy A Ananthakrishnan AN. Influence of environmental factors in the development and outcomes of inflammatory bowel disease. Gastroenterol Hepatol. (2019) 15:72–82. 31011301 PMC6469265

[46] Guo AY Stevens BW Wilson RG Russell CN Cohen MA Sturgeon HC. Early life environment and natural history of inflammatory bowel diseases. BMC Gastroenterol. (2014) 14:216. 10.1186/s12876-014-0216-8 25510175 PMC4300207

[47] Roberts SE Wotton CJ Williams JG Griffith M Goldacre MJ. Perinatal and early life risk factors for inflammatory bowel disease. World J Gastroenterol. (2011) 17:743–49. 10.3748/wjg.v17.i6.743 21390144 PMC3042652

[48] Kostic AD Xavier RJ Gevers D. The microbiome in inflammatory bowel disease:current status and the future ahead. Gastroenterology. (2014) 146:1489–99. 10.1053/j.gastro.2014.02.009 24560869 PMC4034132

[49] Probert CS Jayanthi V Hughes AO Thompson JR Wicks AC Mayberry JF. Prevalence and family risk of ulcerative colitis and Crohn's disease: an epidemiological study among Europeans and South Asians in Leicester-shire. Gut. (1993) 34:1547–51. 10.1136/gut.34.11.1547 8244142 PMC1374420

[50] Halme L Turunen U Helio T Paavola P Walle T Miettinen A. Familial and sporadic inflammatory bowel disease: comparison of clinical features and serological markers in a genetically homogeneous population. Scand J Gastroenterol. (2002) 37:692–8. 10.1080/00365520212511 12126248

[51] Yang H McElree C Roth MP Shanahan F Targan SR Rotter JI. Familial empirical risks for inflammatory bowel disease: differences between Jews and non-Jews. Gut. (1993) 34:517–24. 10.1136/gut.34.4.517 8491401 PMC1374314

[52] Peeters M Nevens H Baert F Hiele M de Meyer AM Vlietinck R. Familial aggregation in Crohn's disease: increased age-adjusted risk and concordance in clinical characteristics. Gastroenterology. (1996) 111:597–603. 10.1053/gast.1996.v111.pm8780562 8780562

[53] Halme L Paavola-Sakki P Turunen U Lappalainen M Farkkila M Kontula K. Family and twin studies in inflammatory bowel disease. World J Gastroenterol. (2006) 12:3668–72. 10.3748/wjg.v12.i23.3668 16773682 PMC4087458

[54] Freud A Beharier O Walfisch A Sergienko R Landau D Sheiner E. Maternal inflammatory bowel disease during pregnancy is not a risk factor for long-term morbidity of the offspring. J Crohns Colitis. (2016) 10:1267–72. 10.1093/ecco-jcc/jjw083 27085078

[55] Inoue M Hasegawa A Hosoi Y Sugiura K. Breed, gender and age pattern of diagnosis for veterinary care in insured dogs in Japan during fiscal year 2010. Prev Vet Med. (2015) 119:54–60. 10.1016/j.prevetmed.2015.02.010 25746927

[56] Hall EJ German AJ Malattia infiammatoria intestinale. In: Steiner JM editor. Gastroenterologia del Cane e del Gatto. Milano: Elsevier (2009). p. 296–311.

[57] Pineton de Chambrun G Body-Malapel M Frey-Wagner I Djouina M Deknuydt F Atrott K. Aluminum enhances inflammation and decreases mucosal healing in experimental colitis in mice. Mucosal Immunol. (2014) 7:589–601. 10.1038/mi.2013.78 24129165 PMC3998638

[58] Geier DA King PG Hooker BS Dórea JG Kern JK Sykes LK. Thimerosal:clinical, epidemiologic and biochemical studies. Clin Chim Acta. (2015) 444:212–20. 10.1016/j.cca.2015.02.030 25708367

[59] Lerner A. Aluminum is a potential environmental factor for Crohn's disease induction: extended hypothesis. Ann N Y Acad Sci. (2007) 1107:329–45. 10.1196/annals.1381.035 17804561

[60] Thompson NP Montgomery SM Pounder RE Wakefield AJ. Is measles vaccination a risk factor for inflammatory bowel disease? Lancet. (1995) 345:1071–4. 10.1016/S0140-6736(95)90816-1 7715338

[61] Davis RL Kramarz P Bohlke K Benson P Thompson RS Mullooly J. Measles-mumps-rubella and other measles-containing vaccines do not increase the risk for inflammatory bowel disease: a case-control study from the vaccine safety datalink project. Arch Pediatr Adolesc Med. (2001) 155:354–9. 10.1001/archpedi.155.3.354 11231801

[62] Shaw SY Blanchard JF Bernstein CN. Early childhood measles vaccinations are not associated with paediatric IBD: a population-based analysis. J Crohns Colitis. (2015) 9:334–8. 10.1093/ecco-jcc/jjv029 25716176

[63] Pineton de Chambrun G Dauchet L Gower-Rousseau C Cortot A Colombel JF Peyrin-Biroulet L. Vaccination and risk for developing inflammatory bowel disease: a meta-analysis of case–control and cohort studies. Clin Gastroenterol Hepatol. (2015) 13:1405–15. 10.1016/j.cgh.2015.04.179 25956840

[64] Latuga MS Stuebe A Seed PC. A review of the source and function of microbiota in breast milk. Semin Reprod Med. (2014) 32:68–73. 10.1055/s-0033-1361824 24390923

[65] Chu DM Meyer KM Prince AL Aagaard KM. Impact of maternal nutrition in pregnancy and lactation on offspring gut microbial composition and function. Gut Microbes. (2016) 7:459–70. 10.1080/19490976.2016.1241357 27686144 PMC5103658

[66] Zhou X Du L Shi R Chen Z Zhou Y Li Z. Early-life food nutrition, microbiota maturation and immune development shape life-long health. Crit Rev Food Sci Nutr. (2019) 59:S30–8. 10.1080/10408398.2018.1485628 29874476

[67] Sallander M Adolfsson J Bergvall K Hedhammar Å Nodtvedt A The effect of early diet on canine atopic dermatitis (CAD) in three high-risk breeds. Open Dermatol J. (2009) 3:73–80. 10.2174/1874372200903010073

[68] Dixon LJ Kabi A Nickerson KP McDonald C. Combinatorial effects of diet and genetics on inflammatory bowel disease pathogenesis. Inflamm Bowel Dis. (2015) 21:912–22. 10.1097/MIB.0000000000000289 25581832 PMC4366276

[69] Indrio F Martini S Francavilla R Corvaglia L Cristofori F Mastrolia SA. Epigenetic matters: the link between early nutrition, microbiome, and long-term health development. Front Pediatr. (2017) 5:178. 10.3389/fped.2017.00178 28879172 PMC5572264

[70] Hall EJ Batt RM. Dietary modulation of gluten sensitivity in a naturally occurring enteropathy of Irish setter dogs. Gut. (1992) 33:198–205. 10.1136/gut.33.2.198 1347279 PMC1373930

[71] Hall EJ Batt RM. Delayed introduction of dietary cereal may modulate the development of gluten-sensitive enteropathy in Irish setter dogs. J Nutr. (1991) 121:S152–3. 10.1093/jn/121.suppl_11.S152 1941212

[72] Hall EJ Batt RM. Differential sugar absorption for the assessment of canine intestinal permeability: the cellobiose/mannitol test in gluten-sensitive enteropathy of Irish setters. Res Vet Sci. (1991) 51:83–7. 10.1016/0034-5288(91)90036-N 1910201

[73] Martinez-Medina M Denizot J Dreux N Robin F Billard E Bonnet R. Western diet induces dysbiosis with increased E coli in CEABAC10 mice, alters host barrier function favouring AIEC colonisation. Gut. (2014) 63:116–24. 10.1136/gutjnl-2012-304119 23598352

[74] Kamada N Kim YG Sham HP Vallance BA Puente JL Martens EC. Regulated virulence controls the ability of a pathogen to compete with the gut microbiota. Science. (2012) 336:1325–9. 10.1126/science.1222195 22582016 PMC3439148

[75] Steinhoff-Wagner J Zitnan R Schonhusen U Pfannkuche H Hudakova M Metges CC. Diet effects on glucose absorption in the small intestine of neonatal calves: Importance of intestinal mucosal growth, lactase activity, and glucose transporters. J Dairy Sci. (2014) 97:6358–69. 10.3168/jds.2014-8391 25108868

[76] Andoh A Bamba T Sasaki M. Physiological and anti-inflammatory roles of dietary fiber and butyrate in intestinal functions. JPEN J Parenter Enteral Nutr. (1999) 23:S70–3. 10.1177/014860719902300518 10483900

[77] Looijer-van LMA Dieleman LA. Prebiotics in chronic intestinal inflammation. Inflamm Bowel Dis. (2009) 15:454–62. 10.1002/ibd.20737 18831524 PMC5148622

[78] Satpute M Annapure U Approaches for delivery of heat sensitive nutrients through food systems for selection of appropriate processing techniques: a review. J Hyg Eng Design. (2013) 4:71–92.

[79] Reddy MB Love M. The impact of food processing on the nutritional quality of vitamins and minerals. In: Jackson LS Knize MG Morgan JN ediors. Impact of Processing on Food Safety. Advances in Experimental Medicine and Biology. Boston, MA: Springer (1999). p. 459. 10.1007/978-1-4615-4853-9_7 10335371

[80] Chassaing B Koren O Goodrich JK Poole AC Srinivasan S Ley RE. Dietary emulsifiers impact the mouse gut microbiota promoting colitis and metabolic syndrome. Nature. (2015) 519:92–6. 10.1038/nature14232 25731162 PMC4910713

[81] National Research Council Nutrient Requirements of Dogs. Washington, DC: The National Academies Press (1974).

[82] Hilton J. Carbohydrates in the nutrition of dog. Can Vet J. (1990) 31:128–9. 17423517 PMC1480633

[83] Coppinger R Coppinger L Dogs. In: A startling New Understanding of Canine Origin, Behavior and Evolution. Prentice, Hall and IBD. New York, NY: Scribner (2001).

[84] Brown S The canine ancestral diet. In: Unlocking the Canine Ancestral Diet: Healthier Dog Food the ABC Way. Wenatchee, WA: Dogwise Publishing (2010). p. 5–11.

[85] Algya KM Cross TL Leuck KN Kastner ME Baba T Lye L. Apparent total-tract macronutrient digestibility, serum chemistry, urinalysis, and fecal characteristics, metabolites and microbiota of adult dogs fed extruded, mildly cooked, and raw diets. J Anim Sci. (2018) 96:3670–83. 10.1093/jas/sky235 29893876 PMC6127788

[86] Ng SC Zeng Z Niewiadomski O Tang W Bell S Kamm MA. Early course of inflammatory bowel disease in a population-based inception cohort study from 8 countries in Asia and Australia. Gastroenterology. (2016) 150:86–95. 10.1053/j.gastro.2015.11.019 26385074

[87] Ellulu MS Patimah I Khaza'ai H Rahmat A Abed Y. Obesity and inflammation: the linking mechanism and the complications. Arch Med Sci. (2017) 13:851–63. 10.5114/aoms.2016.58928 28721154 PMC5507106

[88] Cortese L Terrazzano G Pelagalli A. Leptin and immunological profile in obesity and its associated diseases in dogs. Int J Mol Sci. (2019) 20:2392. 10.3390/ijms20102392 31091785 PMC6566566

[89] Waitzberg DL Ravacci GR Raslan M. Hospital hyponutrition. Nutr Hosp. (2011) 26:254–64. 10.1590/S0212-16112011000200003 21666960

[90] Elsherif Y Alexakis C Mendall M. Determinants of weight loss prior to diagnosis in inflammatory bowel disease: a retrospective observational study. Gastroenterol Res Pract. (2014) 2014:762191. 10.1155/2014/762191 25506359 PMC4259140

